# Social networks and aggressive attitudes: who is who. Scoping review of the scientific production on their relationships

**DOI:** 10.3389/fpsyg.2023.1249907

**Published:** 2023-11-09

**Authors:** Margarita Martín-Martín, José Antonio Bueno-Álvarez

**Affiliations:** Department of Research and Psychology in Education, Complutense University of Madrid, Madrid, Spain

**Keywords:** social networks, moral disengagement, disruptive behavior, aggressiveness, review

## Abstract

**Introduction:**

In the current world, an increasing number of people use social networks as a scenario for socialization, which have come to stay as a part of human development. During this socialization process, violent situations occur all too often, despite their virtuality, and seriously compromises the emotional well-being of the other participants. Based on the work conducted on this subject, the following systematic review aims to establish the state of the art regarding the relationship between moral disengagement, disruptive behavior and emotional intelligence of social network users.

**Method:**

A scoping review is carried out, according to the PRISMA-ScR criteria, consulting the WoS, Scopus, Education database, PsycINFO, PsycARTICLES, PLOS one and ScienceDirect databases, from 2021 up to the present day.

**Results:**

A total of 999 articles related to the research topic were collected, although the result of research responding to the specific search criteria was reduced to 10.

**Discussion:**

The research identified shows that there is a relationship between the level of moral development of social network users and their participation in aggressive online behavior. However, more research is needed, as it has not been demonstrated whether it is the networks that develop or favor the emergence of these attitudes, or simply act as facilitators for their amplified expression.

## 1. Introduction

In recent years, the interest inspired using the internet and social media, and the important impact they have had on people’s lives has inspired many academic publications. Technology is a major part of our society, and this explosion in information and communication technologies has led to radical changes in how we live, at all levels. This impact is even greater among the younger generations, as they more often use social media as the fundamental setting for relating to others.

While ICT offers great benefits, the risks and problems associated with it are currently one of the greatest concerns at a global level (Gini et al., 2014; Lee and Shin, 2017; Kowalski et al., 2018; Zhao and Yu, 2021; Zhu et al., 2021). Cyberbullying is one of the most serious, and its prevalence is increasing (Kowalski et al., 2014; Watts et al., 2017; Fang et al., 2020). This is defined as behavior comprising online aggression against another person, perpetrated by individuals or groups repeatedly and intentionally, with the intention of inflicting distress or harm. It also displays the characteristic of anonymity, concealment, and virtuality that the internet offers, as it is relatively simple for cyberbullies to hide behind screen names or false names (Peter and Petermann, 2018; Wang and Ngai, 2020; Chan et al., 2021; Zhao and Yu, 2021). Although the most common loci for cyberbullying are social media, it can also happen through email, chats, online games, web pages or digital images (Kowalski et al., 2018; Paciello et al., 2020; Zhang and Zhao, 2020). It can take very varied forms, most notably: flaming, or use of vulgar language; trolling, or forcing people to argue through negative communication; denigration, or spreading harmful rumors; masquerade, which consists of hiding one’s true identity; exclusion, which involves removing someone from a group; outing, or revealing private information; cyberstalking or sending offensive messages; harassment, which comprises sending offensive messages; and fraping, which involves publishing inappropriate content in a person’s accounts to make other users believe that the victim has posted it (Shaikh et al., 2020).

Given the above, there is no doubt that cyberbullying is a phenomenon that can have serious consequences for its victims and their environments, and it is regarded as potentially more harmful than traditional bullying thanks to the lack of spatial–temporal boundaries of the internet (Gao et al., 2020). These consequences for the victim can be of various types, including anxiety, depression, substance abuse (Fang et al., 2020), self-harming and even suicide (Kowalski et al., 2014). To sort and systematize the evidence currently available, there have been numerous systematic reviews paying special attention to victims and witnesses or observers of the situation (Zhu et al., 2021). This has left to one side the people who commit this violence, which has not been the subject of exhaustive research in this respect (Shaikh et al., 2020; Imuta et al., 2022). For this reason, it is necessary to explore the factors or circumstances that can contribute to cyberbullying among children and adolescents.

Authors such as Bauman (2010), Perren and Sticca (2011), and Pornari and Wood (2010) have studied how virtual settings can foster a lack of moral engagement with actions done in them because the reaction of the cybervictim is not observed. In these virtual scenarios, the social rules and limits of the offline world can be ignored with fewer immediate consequences. In addition, it is simple to share, retweet and repeat bullying messages online (Zhao and Yu, 2021). Therefore, it is more probable that cyberbullying behavior will be associated with a higher level of moral disengagement (Busching and Krahé, 2015; Zhao and Yu, 2021). The term “moral disengagement,” proposed by Bandura et al. (1996) based on social cognition theory, is defined as a set of self-regulating mechanisms by which individuals justify their immoral behavior to make their actions seem less damaging (Bjärehed et al., 2020; Fang et al., 2020; Marín-López et al., 2020). This process comprises four moral disengagement strategies and eight mechanisms. The first strategy is cognitive restructuring, which includes the mechanisms of euphemism, advantageous comparison, and moral justification. The second strategy is minimization of one’s own role, with mechanisms such as displacement/diffusion of responsibility. The third is ignorance or distortion of consequences, which includes ignoring/distorting consequences, and the last strategy is blaming or dehumanizing the victim, with attribution of blame and dehumanization (Bandura, 1990, 1991, 2016). Given that virtual settings generate feelings of anonymity, impunity and false protection owing to the characteristics of online communication, Runions and Bak (2015) analyzed how the lack of social and emotional signs, diffusion of messages on social media and the role of the media in the diffusion of cases of cyberbullying can facilitate moral disengagement. Moreover, for Zhao and Yu (2021), the virtual world does not feature the same mechanisms as the offline world to establish social norms and supervision and moral evaluation systems, and so it is less likely that people will form a morality influenced by external norms and positive behavior. Numerous studies have indicated that a high level of moral disengagement is related with adolescents with a higher propensity to cyberbully peers (Lazuras et al., 2013; Wang et al., 2017; Orue and Calvete, 2019). Similarly, many studies have also shown that gender significantly moderates the relationship between moral disengagement and cyberbullying perpetration, and the relationship between moral disengagement and cyberbullying was stronger for males than that for females (Kowalski et al., 2014; Wang et al., 2016). Research indicates that boys show fewer moral feelings, such as guilt or empathy, than girls (Bussey et al., 2015). This is partly explained because girls tend to desire more often positive relations with others, so they limit their engagement in cyberbullying behaviors, even when they have higher levels of moral disengagement (Wang et al., 2016).

As these relationships do not always occur, so other mediator variables that influence cyberbullying must be studied. It is for this reason that our aim in this study is to analyze the evidence that underlines the role of moral disengagement in the profiles of cyberbullies, and to identify the association that can appear between moral disengagement (MD) and other psychological variables when perpetrating cyberbullying. In this way, we will arrive at an analysis of determining factors that must be considered when designing and planning specific actions aimed at preventing this type of behavior online.

## 2. Methodology

To achieve the proposed aim, we carried out a scoping review (Arksey and O’Malley, 2005). According to Munn et al. (2022), this type of review has the aim of systematically synthesizing the available evidence, in a particular context, of a subject or field to enable key characteristics or factors related to be identified. To do so, a psychology librarian was consulted to define the search strategies (following Chong et al., 2022), adapting the “Objectives” section of the PRISMA-ScR protocol (Tricco et al., 2018) by replacing PICO with SPIDER, where: S refers to subjects who use social media; P-I is any type of violence exercised through social media; D is a descriptive study; E is moral disengagement; and R is quantitative studies. The SPIDER acronym (Cooke et al., 2012) best meet the review objectives (Marín, 2022). No review protocol was previously published.

### 2.1. Inclusion and exclusion criteria

The inclusion criteria were articles published in open access peer reviewed journals; between 2021 and the present day (given that the most recent systematic reviews on this topic covered the period until then); covering the topic of online violence; specifically, the subject who acts as bully, hater or aggressor; in its aspect of moral disengagement; written in English. In contrast, we rejected results that centered on sports, the workplace, management or leadership, clinical treatments or social programs; studies focused on the victim, witness, disseminator, parents, teachers or other people; works with a single case study, systematic review or meta-analysis methodology; studies in books, book chapters doctoral theses/dissertations, reviews, editorials, conference proceedings, study records, clinical study reports, unpublished manuscripts, or government reports. We did not contact any authors or institutions to identify further sources.

### 2.2. Information sources

The databases consulted were Education Database (via ProQuest), APA PsycARTICLES (via EBSCOhost), APA PsycINFO (via EBSCOhost), ScienceDirect, Scopus and Web of Science. The search engine Google Scholar was also used. Finally, PLOS ONE journal was also consulted, as it is the open access journal that has been publishing the largest number of articles (STM Publishing News, 2017). The search was done during the last week of January 2023.

### 2.3. Search strategy

The descriptors for the search equation were chosen using the previously published systematic reviews as a reference, as well as the terms in the Education Resources Information Center (ERIC) and European Education thesauruses. The search equation developed was: (((“moral disengagement” OR “moral dilemma*” OR “moral reasoning” OR “moral development” OR “prosocial moral reasoning” OR “unethical decision-making”) AND (“virtual on-line social network*” OR “on-line social network” OR “social media” OR “social network*” OR “cyber*” OR “on-line interaction*” OR “digital identit*”)) OR (“moral disengagement through technolog*” OR “problematic social network* site* use*” OR “on-line morality” OR “on-line disruptive behavior*”)). However, after a first pilot search, owing to the small and insignificant number of results found, and the limitations of the search engines of the databases consulted, we followed the recommendation of Bramer et al. (2018) and reformulated the search equation, which finally took the form of: (“moral development” OR “moral disengagement”) AND (“social network” OR “cyber”).

### 2.4. Selection and filtering

**Figure 1:**
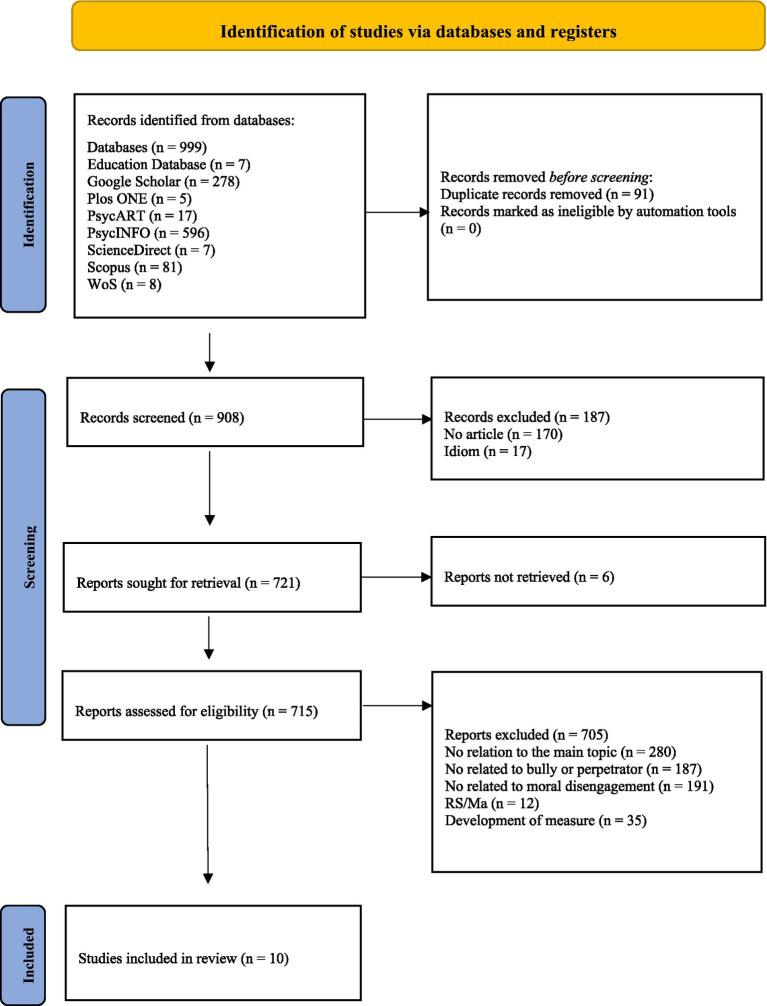
Flow diagram of the study selection process.

Once the search had been performed, after eliminating duplicate records, the inclusion and exclusion criteria were progressively applied, following the repeated reading and examination of the title, abstract and text of the records identified (6 articles were unreachable). The results of these successive analyzes are shown in the flow chart in Figure 1, drawn up based on the PRISMA 2020 standards (Page et al., 2021). To avoid bias in the final selection of registers, given that the study only has two authors (Khalil et al., 2021), we followed the procedure used by Dinsmore and Fryer (2023) and, after eliminating duplicates, we started to code independently 100 records from the total identified for inclusion or exclusion to establish whether we could reliably judge in accordance with the criteria mentioned. This independent coding revealed a level of agreement of 95% between the authors. After resolving disagreements in the independent coding and given the high level of agreement between the evaluators, the second author coded the remaining records independently for inclusion/exclusion from the final set of studies. As a result, 10 studies were selected for analysis.

### 2.5. Evaluation of bias of the publications

Owing to the nature and purpose of the systematic review methodology used, as well as the selection criteria chosen, we did not evaluate the quality of the primary sources (Munn et al., 2018).

### 2.6. Data extraction

The relevant information was extracted from each of the articles finally included, referring fundamentally to sample characteristics, measurement instruments used, other study variables, results, possible biases/limitations. The data extracted were compiled in a Microsoft® Excel 2010 spreadsheet for identification and classification, as well as to analyze and synthesize the information, in accordance with the objectives of the review.

## 3. Results

Table 1 summarizes the principal results. It shows the articles finally selected ordered alphabetically and numbered.

**Table 1:** Study characteristics.

Study author(s), (year)	*N*	Age/Year	Female (%)	Country	Moral Disengagement Measure (MD)	Other variables	Results
Gajda et al. (2022)	251	18–60 (*M* = 28.54)	72.6	Poland	The Mechanisms of moral Disengagement scale by Bandura et al. (1996)	Dark triad (DT) Cyberbullying (CB)	Positive associations between DT and antisocial online behavior. Sadism, Machiavellianism, and psychopathy were positively associated with cyberbullying and cybervictimization. Individuals who have experienced cybervictimization can become offenders on the Internet due to their revenge motivation. Machiavellianism and sadism were correlated with moral disengagement.
Lee and Jang (2022)	400	*M* = 22.65	50	South Korea	The Mechanisms of Moral Disengagement Scale by Bandura et al. (1996). Adapted into Korean and validated by Youn (2014), (Unpublished doctoral dissertation)^1^.	Type D personality Cyber-aggression	Male students showed greater moral disengagement than female students. Male students showed greater cyber aggression in a non-anonymous context, and according to Type D personality in an anonymous context. In a non-anonymous context, cyber aggression was not significantly correlated with the Type D personality but positively correlated with MD. In an anonymous context, cyber aggression was positively correlated with Type D personality and with MD. No significant correlation between cyber aggression and Type D personality in a non-anonymous context.
Llorent et al. (2021)	2,535	9–16	50.5	Spain, Poland	The Mechanisms of Moral Disengagement Scale by Bandura et al. (1996). Moral emotions scale by Álamo et al. (2020)	Bullying and cyberbullying Social and emotional competences Empathy	Polish students scored higher in bullying and cyberbullying victimization and perpetration, in both levels. Polish students also scored higher in moral disengagement. No significant differences in moral emotions in primary education, while in secondary education, Spanish students scored significantly higher. In social and emotional competencies, Spanish students scored higher than Polish students in secondary while Polish students scored higher in primary education.
Luo and Bussey (2022)	563	Years 7,9 (*M* = 12.73/14.72)	39.4	Australia	16 items from the Mechanisms of Moral Disengagement Scale by Bandura et al. (1996)	Cyberbullying roles	MD partially mediated the link between cyber victimization, cyber bystander and cyber perpetration. MD facilitates victims to engage in bullying despite having been previously victimized themselves. Witnesses to cyberbullying tended to report higher levels of MD, which was also linked with greater levels of perpetration.
Maftei et al. (2022)	509	11–67 (*M* = 21.33, SD = 12.37)	60.3	Romania	A modified version of the Mechanisms of Moral Disengagement Scale by Bandura et al. (1996)	Compulsive internet use Cyberbullying	Teenagers scored significantly higher than adults on moral disengagement, compulsive internet use, and cyberbullying through fake news. Adults engage in cyberbullying to a lesser extent than adolescents, depending on how morally disengaged they are.
Mascia et al. (2021)	174	11–17	-	Italy	A 32 item Italian version of the Mechanisms of Moral Disengagement Scale by Bandura et al. (1996)	Empathy Victim’s experience representations (VER)	Positive correlations between empathy and the perceived consequence of CB on the victim. Positive correlation between empathy and perceived predisposing factors for victimization. Significant negative correlation between empathy and MD and with the victim’s reactions to CB. VER: positive correlation with the victim’s reactions and the perceived predisposing factors for victimization. Low cognitive empathy increased the probability of perpetration. Low perception of consequences increased the probability of belonging to the bully group, to the group of individuals having had both experiences (bully/victim), and to the group of individuals who have never experienced CB.
Nocera et al. (2022)	404	18–29 (*M* = 25.16, SD = 2.76)	59	United States	Moral Disengagement Measure by Detert et al. (2008)	Demographics Cyberbullying perpetration Triarchic psychopathy Sadistic tendencies Aggression	Sadistic traits, psychopathic traits, and trait anger predicted cyber aggression perpetration. MD partially mediated these relationships, as a mechanism through which trait anger and dark personality traits are connected to cyber aggression. Trait anger as a predictor of cyber aggression.
Rodríguez-deArriba et al. (2023)	1,160	12–17 (*M* = 14.25, SD = 1.35)	52.7	Spain	Mechanisms of Moral Disengagement Scale by Bandura et al. (1996)	Online control and online jealousy Socio-emotional competence	Girls: higher levels of online jealousy and online control than boys. Boys: higher levels of MD. No significant gender differences in socio-emotional competence. MD: strong moderator between online jealousy and online control in both genders Jealous adolescents who justified violence were more likely to exercise control over their partners in the online context.
Yang et al. (2022)	2,407	11–16 (*M* = 12.75)	50.2	China	Mechanisms of Moral Disengagement Scale by Bandura et al. (1996)	Peer pressure Cyberbullying perpetration. Family socioeconomic status.	Reciprocal relationships between peer pressure and cyberbullying perpetration. Peer pressure predicted cyberbullying perpetration through MD. Perceived pressure from peers is a sufficient moral reason for adolescents’ self-justification of MD. The relation between peer pressure and moral disengagement was more robust for adolescents with low family socioeconomic status. Boys had more cyberbullying perpetration than girls.
Zhang et al. (2022)	521	11–20 (*M* = 14.01)	49.1	China	Moral Disengagement Scale by Moore et al. (2012)	Dark triad Cyber aggression	DT was positively related to CA among adolescents. MD mediated the connection between DT personality traits and adolescents’ *CA.* DT personality traits are more strongly associated with CA for women than for men.

1 Youn, H. (2014). *Study on unethical decision making of people with psychopathic tendency: Focused on moral emotion and moral disengagement* [Unpublished doctoral dissertation]. Catholic University of Korea.

### 3.1. Samples

Of the 10 articles selected, 70% use Western samples from a variety of countries such as Australia (Luo and Bussey, 2022), Spain (Llorent et al., 2021; Rodríguez-deArriba et al., 2023), Italy (Mascia et al., 2021), Poland (Llorent et al., 2021; Gajda et al., 2022), Romania (Maftei et al., 2022) and the United States (Nocera et al., 2022). The other studies used samples from China (Yang et al., 2022; Zhang et al., 2022) and South Korea (Lee and Jang, 2022). The samples mainly comprise adolescent subjects and secondary education or baccalaureate students [60% in the study by Maftei et al. (2022)]; in almost all cases they are people aged under 22 years, except in the studies by Gajda et al. (2022), Lee and Jang (2022), and Nocera et al. (2022), with means that are higher but still under 30 years. There are also more female subjects in 80% of the studies.

### 3.2. Variables studied

Moral disengagement is measured as the principal or dependent variable in all the articles. The independent or predictor variables that have been considered include personality disorders [type D and Dark Triad (Gajda et al., 2022; Lee and Jang, 2022; Nocera et al., 2022; Zhang et al., 2022)], risks on social media [cyberbullying (Llorent et al., 2021; Luo and Bussey, 2022; Maftei et al., 2022; Nocera et al., 2022; Yang et al., 2022), cyberaggression (Lee and Jang, 2022; Zhang et al., 2022), online control and pathological jealousy (Rodríguez-deArriba et al., 2023), compulsive use (Maftei et al., 2022)], socio-emotional skills (Llorent et al., 2021; Rodríguez-deArriba et al., 2023), empathy (Llorent et al., 2021; Mascia et al., 2021) and sociodemographic characteristics (Nocera et al., 2022; Yang et al., 2022).

### 3.3. Moral disengagement measurements

To evaluate MD, almost all the studies use the original version of the Mechanisms of Moral Disengagement Scale (MMDS), by Bandura et al. (1996), either the complete version or some items from it (Luo and Bussey, 2022), or they use it in combination with others [Moral Emotions Scale (MES) by Álamo et al. (2020) and Llorent et al. (2021)]. The study by Nocera et al. (2022) uses the Moral Disengagement Measure of Detert et al. (2008), which is developed from MMDS. Many works do not specify the version or adaptation they have made to apply it to their samples; only the studies by Maftei et al. (2022) and Mascia et al., 2021 state this, limiting themselves to stating that they have modified it or adapted it into Korean. Only one study (Zhang et al., 2022), used a different scale: the Moral Disengagement Scale (MDS) by Moore et al. (2012).

### 3.4. Analysis of mediator effects

MD is directly related to the traits of Machiavellianism, sadism, psychopathy, and anxiety (type D personality), which in turn are associated with antisocial online behaviors, such as cyber-aggression and cybervictimization (Gajda et al., 2022; Zhang et al., 2022), being the relationship MD-type D personality stronger in women (Zhang et al., 2022). For several authors, it is so evident that DM is a moderating variable (Luo and Bussey, 2022; Nocera et al., 2022) that in situations of anonymous cyberbullying, the DM- type D personality relationship is more evident, but when subjects are identifiable, perpetration of antisocial behavior only depends on the degree of MD of the individual (Lee and Jang, 2022; Nocera et al., 2022).

Moreover, the level of MD acts as a moderator of the probability that a victim of cyber-aggression will subsequently act as an aggressor (Luo and Bussey, 2022), which turns out to be quite frequent because of the desire for revenge that has been generated (Gajda et al., 2022).

Regarding to gender, males have higher levels of MD and are more prone to cyber-aggression (Lee and Jang, 2022; Yang et al., 2022), especially if they are teenagers (Rodríguez-deArriba et al., 2023). However, and thanks to the development of MD achieved over time, men do not have to maintain the trend toward cyberbullying behaviors (Maftei et al., 2022).

MD is affected when witnessing cyberbullying situations (Luo and Bussey, 2022), to the extent that witnesses find justifications for this behavior and are pressured by the group to engage in it (Yang et al., 2022) when seeing the lack of consequences, although this predisposition is mediated by the witnesses’ degree of empathy toward the victim (Mascia et al., 2021). Group pressure is more apparent when the subject is an adolescent and come from low-income family settings (Yang et al., 2022).

MD also influences online control and surveillance of electronic devices in partner relationships between adolescents (Rodríguez-deArriba et al., 2023), without significant differences between male and female subjects, although the latter are more prone to do this online.

## 4. Discussion and conclusions

Our aim in this study was to analyze the evidence that underlines the role of moral disengagement in cyberbullies and to determine the association that might occur between moral disengagement and other psychological variables when perpetrating cyberbullying. We have made progress in the analysis of the determining factors that must be considered when designing and planning specific actions aimed at preventing these behaviors online.

First, we found that there is little literature on this subject, although the studies that do exist all point in the direction of moral disengagement having an important role in the predisposition to commit cyberbullying. Perpetrators obtain higher scores in moral disengagement, independently of the context in which the studies are performed. As we verified in our review, research indicates that males show higher levels of MD than females, which is consistent with previous studies (Bandura et al., 1996; Bussey et al., 2015; Wang et al., 2016; Zhao and Yu, 2021).

Although most studies show that the tendency to commit acts of cyberbullying is greater in boys, some studies already indicate that the proportion of cyberbullying happened in cyberspace is similar between males and females, and the difference lies in the way in which cyberbullying is committed (Kowalski et al., 2018; Martínez-Pecino and Durán, 2019; Wang et al., 2019). Some researchers found that girls usually use emails or chat rooms for cyberbullying (Zych et al., 2019), while boys often employ text messages or online games for cyberbullying (Wang et al., 2016; Romera et al., 2021). In these situations, the disinhibitory effect associated with anonymity is of great importance. Suler (2004) found out that people could openly display their aggression in cyberspace, whereas, owing to social desirability, it was suppressed in the real world.

Secondly, it has been found that, as well as the moral disconnection, there is a series of psychological variables that are directly related with the probability of committing cyberbullying. Shaikh et al. (2020) performed a systematic review in which they found that personality, stress, anxiety, depression, emotional intelligence, revenge, loneliness, frustration, self-esteem, aggression, empathy, antisocial behavior, insecurity, internalization and jealousy are crucial in the phenomenon of cyberbullying. In relation to our results, previous studies confirm this association in both the empathy variable and socio-emotional competence. Schultze-Krumbholz et al. (2018) demonstrated the role of empathy as a protective factor against cyberbullying, while Mascia et al. (2021) noted the importance of social keys as activators of processes of empathy, as certain elements of language are absent in online settings, which can facilitate hostile and aggressive attitudes toward others in bullies.

Our results also indicated that the dark triad personality, namely traits of Machiavellianism, psychopathy, and narcissism, is positively related to cyberbullying. This agrees with the study by Nocera et al. (2022), which found that psychopathic traits are the most important ones in aggression, violent behavior, bullying, moral foundations and cyber-aggression owing to a lack of empathy and excess impulsiveness. Common characteristics of the three traits in the triad are disdain for social values, aversive social insensitivity, a history of being abusive, lack of empathy, disagreeableness, and impulsivity (Zhang et al., 2022).

In the light of our analysis, we believe that the role of education is especially important in the specific actions that must be taken to reduce the likelihood of cyberbullying actions being perpetrated against any person, especially in the case of children and adolescents. As Llorent et al. (2021) and Marín-López et al. (2020) observe, a holistic approach from schools is required to involve the curriculum and the planning of actions, without neglecting the role of families in the development of children and adolescents.

Since the relationship between MD and cyberbullying is evident, it is necessary to work comprehensively, from education, on the formation of moral values that allow understanding the reasons why cyberbullying cannot be committed in any of its forms. It is important that there is coherence between what is taught at school, at home and what is seen in our society, so it is everyone’s responsibility to foster in the new generations both emotional intelligence, for the management of emotions, and social skills, especially in subjects who present type D personality traits. At the educational level, both guidance departments and tutorial action should have plans that include this type of training from very early stages. For these actions to be truly effective, we suggest including in the programs analysis of profiles that make more in-depth prevention or even intervention. In relation to gender, since men’s peer relationships play a decisive role in non-moral behavior, positive relationships based on respect for others, with clear moral boundaries, should be encouraged.

## 5. Limitations

The most significant limitation that might affect this study is the systematic review methodology used (scoping review). While this does enable a panoramic overview of the state of the question in accordance with the objective set, it cannot go into detail on the directionality of the relationships established between the different variables studied. To do so, it would be advisable to use another of the various systematic review methodologies (Sutton et al., 2019). For their part, the conclusions we reached should be limited to Western study populations, which are predominant in the studies reviewed, being used in 70% of them, with samples that are not necessarily representative as most of them were convenience or accidental, and with certain prior biases (predominance of women, subjects aged under 30 years). Similarly, the data to establish the relationships identified were obtained using self-report techniques, which do not always achieve reliable results to define precisely the role that the variables studied play, and which, therefore, are accompanied by other more comprehensive ones (for example, anecdotal, guided interview, encounter groups).

## Data availability statement

The raw data supporting the conclusions of this article will be made available by the authors, without undue reservation.

## Author contributions

MM-M and JB-Á contributed to conception and design of the study, the organization of the database, the elaboration and revision of the manuscript, and finally read and approved the submitted version. All authors contributed to the article and approved the submitted version.
